# A novel deep learning-based grading system for assessing breast arterial calcification on mammograms, as an independent risk factor for predicting adverse cardiovascular events

**DOI:** 10.1007/s11547-026-02251-8

**Published:** 2026-06-29

**Authors:** Mu’ath Ibrahim, Patrick C. Brennan, Mo’ayyad E. Suleiman, Mary Rickard, Clare Arnott, Jennifer Y. Barraclough, Seyedamir Tavakoli Taba, Ziba Gandomkar

**Affiliations:** 1 The University of Sydney, Camperdown, NSW 2006 Australia; 2 The George Institute for Global Health, Level 18, International Towers 3, 300 Barangaroo Avenue, Barangaroo, NSW 2000 Australia

**Keywords:** Breast arterial calcification, Mammography, Deep learning, Cardiovascular risk prediction, Women

## Abstract

**Purpose:**

To develop a deep learning-based framework for automated detection and grading of breast arterial calcification (BAC) on mammograms, and to evaluate its association with major adverse cardiovascular events (MACE).

**Material and methods:**

This retrospective case–control study used two datasets: SegModel (1270 mammograms with BAC annotations) and MACEPred (3190 mammographic cases of women with MACE, plus 6458 controls). A U-Net segmentation model and Cox proportional hazards models were used to assess adjusted hazard ratios (HRs) for four BAC grading strategies: binary categorization, area-based grading (none, mild, moderate, severe), intensity-based grading, and a combined approach.

**Findings:**

The U-Net attained a Jaccard similarity coefficient of 0.582, accuracy of 0.996, precision of 0.801, F1 score of 0.756, and recall of 0.716 in segmenting BAC. The presence of BAC was associated with an adjusted HR of 1.16 (95% CI 1.10–1.23). For area-based grading, the HRs for mild, moderate, and severe grades were 1.13 (95% CI 1.06–1.20), 1.30 (95% CI 1.18–1.45), and 1.58 (95% CI 1.15–2.18), respectively. Intensity-based grading showed respective HRs of 1.08 (95% CI 1.00–1.17), 1.10 (95% CI 1.01–1.20), and 1.18 (95% CI 1.09–1.28). The combined approach demonstrated respective HRs of 1.102 (95% CI 1.04–1.17), 1.249 (95% CI 1.14–1.37), and 1.654 (95% CI 1.30–2.10) for mild, moderate, and severe grades.

**Conclusion:**

Our study presents a novel automated framework for BAC assessment that provides independent insights into cardiovascular risk in women. Combined area and intensity grading reflected increasing MACE risk across BAC severity levels, although its incremental predictive improvement was limited.

**Supplementary Information:**

The online version contains supplementary material available at https://doi.org/10.1007/s11547-026-02251-8.

## Introduction

Cardiovascular disease (CVD) remains the leading cause of mortality among women worldwide, surpassing all forms of cancer combined [1, 2]. This persistent burden is shaped by a combination of biological sex-specific factors, such as hormonal influences and vascular physiology, as well as systemic disparities in risk assessment and treatment [3, 4]. Women are frequently underrepresented in cardiovascular research and may exhibit atypical clinical presentations, contributing to diagnostic delays and inconsistent implementation of preventive interventions [5]. Furthermore, a considerable proportion of coronary events in women arise in the absence of conventional risk factors, highlighting the urgent need for refined, gender-sensitive risk stratification models [2, 6].

Breast arterial calcification (BAC), often found incidentally during mammograms, results from calcification to the tunica media of the arterial wall. Its clinical significance was identified in 1980 when linked to diabetes [7], and studies since suggest a positive association between BAC and conditions like CVD and coronary artery disease CAD [8–10], including the presence of atherosclerosis on computed-tomography coronary angiography [10]. A recent cohort study of 5,059 postmenopausal women undergoing mammography confirmed these associations [11].

BAC assessment by radiologists is normally a manual process, followed by a present or absent categorization. This binary classification is prone to intra- and inter-reader variability [8] and fails to capture the full range of BAC appearances. Multiple grading scales (e.g., 3-point, 4-point, 12-point [10]) have been proposed, but the lack of standardized criteria for BAC quantification is a significant challenge. Manual assessment is also time-consuming and may not always be feasible due to limited expert availability [8].

Recent studies have explored deep learning (DL) approaches to assess BAC from digital mammograms, demonstrating DL’s ability to segment BAC at levels comparable to or exceeding human experts [12–14]. However, existing models are trained on limited annotated images, often from a single vendor, raising questions about their generalizability and these models have yet to be validated as independent risk predictors. Furthermore, segmented areas are not translated into an easy-to-communicate grading system that reflects incremental risk changes and potentially nonlinearity in the relationship between BAC and cardiovascular risk. Additionally, the intensity of BAC areas varies, and it is unclear if combining segmentation area with intensity would enhance the grading system.

In this study, we propose a novel deep learning framework that automatically detects and segments BAC on digital mammograms and grades its severity based on both area and intensity. The goal is to offer a reproducible, scalable, and cost-effective tool for cardiovascular risk assessment in women undergoing routine breast cancer screening. We evaluated whether incorporating intensity into area-based grading improves risk prediction by comparing four grading approaches, binary, area-only, intensity-only, and combined, within multivariable-adjusted models for MACE.

## Material and methods

**Fig. 1:**
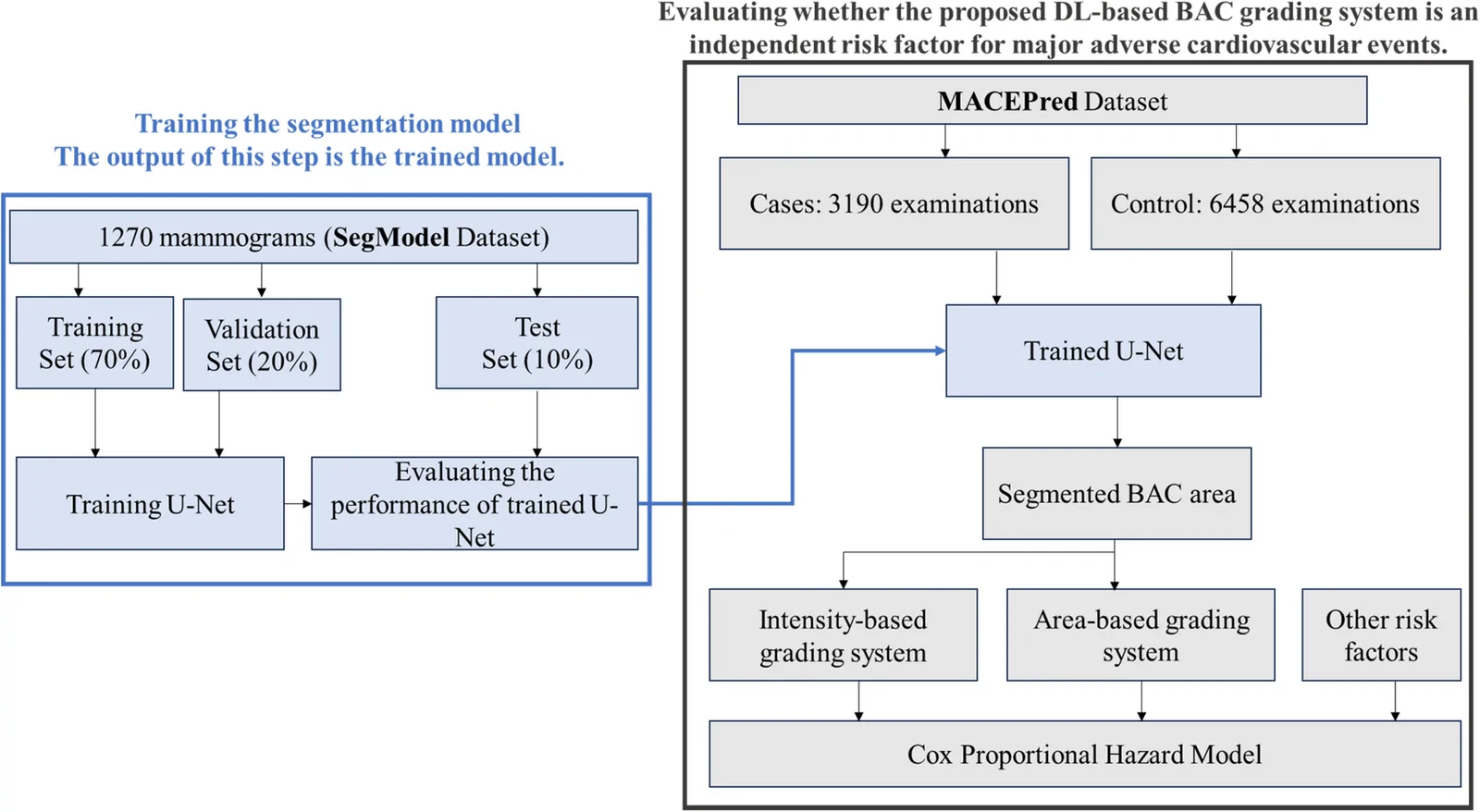
Overview of the study methodology illustrating dataset utilization, segmentation model construction, and integration into Cox proportional hazard analysis for evaluating the DL-based BAC grading

This retrospective case–control study was conducted in accordance with institutional ethical standards and received approval from the relevant Human Research Ethics Committee. All participants had provided informed consent for the use of their imaging and clinical data for research purposes. Figure 1 illustrates the datasets (i.e., SegModel and MACEPred Datasets) utilized in this study. The study steps involved constructing the BAC segmentation model, and subsequently, employing this model to establish the Cox proportional hazards model, thus assessing the efficacy of the proposed DL-based BAC grading framework.

### Dataset

As shown in Fig. 1, we utilized two non-overlapping datasets in this study: the SegModel Dataset and the MACEPred Dataset. Both datasets were sourced from the Lifepool cohort, a large Australian population-based research resource comprising mammographic screening data from more than 50,000 women participating in BreastScreen Australia, with informed consent for linkage to lifestyle, medication, hospitalization, and mortality data. The SegModel Dataset was employed for generating and evaluating the performance of the U-Net in identifying and segmenting BAC areas, while the MACEPred Dataset was used to assess whether the proposed grading system offers an independent risk assessment for MACE. To avoid data leakage, the two datasets were constructed to be mutually exclusive at the participant level, such that no individual or screening examination contributed to both datasets**.** This design ensured that model development and outcome analysis were fully separated, minimizing the risk of biased estimates.

#### SegModel dataset

To construct the SegModel Dataset, an initial random sampling of mammographic images was conducted from the Lifepool archive without identifying BAC presence. However, preliminary experiments showed that images without visible BAC provided limited training value and introduced class imbalance, hindering model convergence. As a result, a medical imaging scientist with expertise in mammography manually selected 1270 images that clearly demonstrated the presence of BAC. While this targeted approach shares some similarity with previous studies such as [12], which also focused on BAC-positive cases, our strategy emphasized vendor diversity to enhance generalizability.

To ensure diagnostic accuracy, all images were independently reviewed by a senior radiologist with over 30 years of experience. BAC regions were manually annotated using Label Studio software [15]. All BAC annotations were reviewed by a senior radiologist, and a second senior radiologist performed an additional quality assurance review to confirm the clinical plausibility and acceptability of the annotated BAC regions for use as training supervision. This review was consensus-based and intended as quality control rather than a formal multi-reader agreement study; therefore, interobserver agreement statistics were not computed for this dataset.

#### MACEPred dataset

The dataset comprises 3190 mammographic cases from women who experienced extended major adverse cardiovascular events (MACE) and from 6458 women who had no such history (controls). These were matched based on their ages and the types of mammography units used for image acquisition, resulting in a case–control ratio of approximately 1:2. Since both mammography unit type and women's ages can alter mammographic textural appearances, we ensured diverse representations in both case and control subsets. Each of the SegModel and MACEPred datasets included screening examinations obtained from 11 different types of mammography units produced by seven vendors, namely Agfa, Fuji, Hologic, Konica Minolta, Philips, Sectra Imtec Ab, and Siemens. By integrating diverse data sources comprehensively, we were able to evaluate the generalizability and performance of our DL-based grading system across various settings. Each screening examination comprised four images: right and left mediolateral oblique (MLO) views and right and left craniocaudal (CC) views. Recruitment of all mammographic cases occurred through the Lifepool project, encompassing women participating in BreastScreen Australia who provided consent to enroll in this project. For each participant, body mass index (BMI), alcohol consumption, and current and former smoker status were collected using a survey. Using data linkage to national dispensing claims (Pharmaceutical Benefits Scheme; PBS), we obtained indicators of medication use in categories relevant to cardiometabolic risk and cardiovascular treatment, including therapies for diabetes and hypercholesterolemia, beta-blockers, calcium channel blockers, angiotensin-converting enzyme (ACE) inhibitors, angiotensin II receptor blockers, and antiplatelet agents. These variables were included as pragmatic proxies for treated risk factors and healthcare contact in the absence of direct clinical measurements (e.g., blood pressure and lipid levels); however, dispensing data do not provide definitive diagnoses, and medication use may reflect both risk factor management and underlying disease severity. The use of routinely captured dispensing data also supports scalable implementation without additional participant questionnaires. Women with evidence of established cardiovascular events prior to the index mammogram were excluded to reduce prevalent disease effects at baseline.

The MACEPred cohort, with an average age of 63.7 ± 7.8 years, the cohort was monitored over a period of 6.09 years on average. The conditions classified MACE included angina pectoris, acute and subsequent myocardial infarction, current complications following acute myocardial infarction (ICD-10 code I23*), other acute ischemic heart diseases (ICD-10 code I24*), chronic ischemic heart disease, cardiac arrest, heart failure, cerebral infarction (stroke, unspecified as hemorrhage or infarction), occlusion and stenosis of precerebral arteries (not resulting in cerebral infarction), occlusion and stenosis of cerebral arteries (not resulting in cerebral infarction), atherosclerosis, aortic aneurysm and dissection, other aneurysms and dissections, other peripheral vascular diseases, arterial embolism and thrombosis, and other disorders of arteries and arterioles. Additionally, any cardiovascular cause of death recorded in the National Death Index was considered a MACE. Further details on the specific ICD-10 codes and outcome groupings used to define MACE are provided in Supplementary Table 1. The characteristics of women included in the study are presented in Table 1.

**Table 1:** Characteristics of women included in the study

Characteristic	Case (*N* = 3190)	Control (*N* = 6458)
Age	63.2 ± 7.6^x̄^	63.9 ± 7.9^x̄^
BMI	26.5 ± 9.9^x̄^	25.6 ± 8.3^x̄^
If had a drink last year	2463 (77%)	5262 (81%)
Being a current smoker	255 (8%)	287 (4%)
Being an ex-smoker	1172 (37%)	2271 (35%)
Presence of BAC	949 (30%)	1652 (26%)
*Medications usage*		
Medications for diabetes	321 (10%)	280 (4%)
Medications for high cholesterol	1252 (39%)	1784 (28%)
Beta blockers	284 (9%)	240 (4%)
Calcium channel antagonists	360 (11%)	409 (6%)
ACE inhibitors	754 (24%)	921 (14%)
Angiotensin II antagonists	825 (26%)	1070 (17%)
Antiplatelets	981 (31%)	936 (14%)

For all variables, the number of cases and their corresponding percentage of the total are presented, except for those marked with x̄, where the mean ± standard deviation is reported

### Training the segmentation model

We chose the U-Net architecture for our base model, given its reputation for effectiveness in biomedical image segmentation [16]. The network employs a four-level encoder–decoder structure with skip connections that preserve spatial detail while integrating multi-scale contextual information. Each encoder block consists of two 3 × 3 convolutional layers followed by rectified linear unit (ReLU) activation and 2 × 2 max-pooling, while the decoder mirrors this structure using transposed convolutions and skip connections for feature reconstruction. 70% of the manually annotated images dataset was used for training (comprising both bilateral and unilateral annotations), 20% for validation, and the remaining 10% for testing. All images were resized 1024 × 1024 before feeding to U-Net. We set a learning rate of 0.001 and used custom loss function that blended binary cross-entropy and Jaccard loss as the loss function, optimized using the Adam optimization algorithm. We included both bilateral and unilateral annotations in our training dataset to provide our model with a comprehensive understanding of breast anatomy and BAC appearances, and to further enrich the model's understanding by offering global context. This approach aligns with clinical practice, where radiologists often consider information from both breasts when assessing mammograms.

To simulate variations in image acquisition conditions across different mammography units, intensity-based augmentation was applied during training. Specifically, random adjustments to brightness (± 20 percent), saturation (± 30 percent), and hue (± 25 percent) were performed in the HSV color space. Geometric augmentations such as rotation or translation were deliberately omitted to preserve the anatomical orientation of standard mammographic views (CC and MLO), as breast arterial calcifications are orientation dependent. Augmentation was applied exclusively to the training set to improve robustness without influencing validation or test performance.

To address class imbalance, the segmentation model was trained exclusively on BAC-positive images. This design choice reflects the primary aim of the model, which was pixel-level segmentation rather than case-level classification of BAC presence. Images without visible BAC provide limited supervision for learning calcification morphology and were found to hinder model convergence during preliminary experiments. Similar training strategies have been adopted in prior BAC segmentation studies, including SCU-Net by Guo et al. [12], as well as in our previously published work using the same architecture [17].

The training dataset included both bilateral and unilateral cases, covering the two standard mammographic views: CC and MLO. This ensured a comprehensive representation of breast anatomy and BAC appearances and reflects routine clinical practice, where radiologists assess both breasts and all standard views during interpretation.

Model performance was evaluated using the Jaccard Similarity Coefficient, accuracy, precision, recall, and F1 score. The Jaccard index quantifies segmentation overlap by measuring the intersection over union between predicted and ground-truth masks. The F1 score, calculated as the harmonic mean of precision and recall, captures the balance between sensitivity and specificity, which is particularly important for detecting small structures such as BAC.

### Grading breast arterial calcifications severity

Four different strategies for translating the area of segmented BAC into a grading system were considered. As is stated in “Material and methods”–“Discussions” sections, the BAC grades were fed into a Cox proportional hazards model and categorizing cases using a grading system can simplify the model, making it easier to interpret and communicate. Using a grading system to categorize cases rather than using the total area of BAC as a continuous variable could also help with handling nonlinearity in the relationship between BAC severity and MACE risk and can mitigate the impact of extreme values (i.e., outliers) by grouping them into broader categories.

The first strategy involved binary categorization of cases. Here, the percentage of breast occupied by BAC was extracted, and if the BAC area was below 0.002 of the total breast area, the case was classified as BAC absent; otherwise, it was considered present. This cutoff was determined through a preliminary sensitivity analysis in which multiple candidate thresholds were evaluated. For each threshold, BAC presence was defined at the participant level using the maximum segmented BAC area, and Cox proportional hazards models were fitted to estimate the association with major adverse cardiovascular events (MACE). Although very low thresholds were statistically associated with MACE, these classified a large proportion of women as BAC-positive and were considered likely to reflect minimal or noise-level segmentations. A threshold of 0.002 was therefore selected as the lowest cutoff associated with a stable and clinically meaningful increase in hazard across adjacent thresholds (Supplementary Fig. 1 and Supplementary Table 5). This threshold was subsequently used to define BAC presence and grading categories in all analyses.

The second strategy introduced a four-scale BAC severity grading system based solely on area. This system yielded four grades: absent (representing BAC area below 0.002 of the total breast), mild (indicating cases with a BAC area between 0.002 and 0.01 of the total breast area), moderate (encompassing cases with a BAC area between 0.01 and 0.025 of the total breast area), and severe (indicating cases with a BAC area exceeding 0.025 of the total breast area). Each grade denoted an increasing severity of BAC presence.

The third strategy relied on the average intensity of the segmented BAC area. Average intensity provides a pixel-based estimate of radiographic brightness and may reflect the physical characteristics of calcified deposits. For each case, the mean pixel intensity within the segmented BAC regions was calculated. To account for systematic differences in image intensity across mammography unit types, these values were normalized using z-score normalization by subtracting the unit-specific mean and dividing by the corresponding standard deviation. The normalized average intensity values were then categorized into four groups. BAC absence was defined using an area threshold below 0.002 of the total breast area and served as the baseline category, as negligible calcified area generally corresponds to minimal radiographic signal and renders intensity measurements unreliable. Among BAC-positive cases, the normalized average intensity distribution was divided into tertiles, with cut-points at 0.409 (33rd percentile) and 1.341 (66th percentile), defining mild (≤ 0.409), moderate (> 0.409 to ≤ 1.341), and severe (> 1.341) intensity-based BAC categories.

The fourth strategy combined both area and intensity assessments. Building upon the area-based and intensity-based grading systems, this approach aimed to establish a unified grading system considering both factors simultaneously. The area-based grade served as the baseline, with adjustments (i.e., uprating) made based on intensity-based grades. For instance, if the area-based BAC grade was “mild” and the intensity-based grade suggested “mild,” the overall grade remained “mild.” However, if the intensity-based grade instead suggested “moderate” or “severe,” the overall grade of mild (from the area measure) was upgraded to “moderate.” Similarly, if the area-based BAC grade was “moderate” but the intensity-based grade was “severe,” the overall grade was assigned as “severe,” one level higher than the area-based grade. Otherwise, it remained “moderate.” Finally, if the BAC grade based on area was “severe,” regardless of the intensity grading, a grade of “severe” was assigned to the case. The area-based grade was selected as the baseline to define severity progression, with intensity-based grading incorporated according to predefined uprating rules. Other approaches, such as considering the maximum value of two grading approaches or including both grades, were also explored, with results presented in the Supplementary Materials.

### Statistical analysis

Four Cox proportional hazards models were constructed, each corresponding to one of the four BAC grading strategies: binary, area-based, intensity-based, and combined. Each model included BAC grade and all available cardiovascular risk factors to estimate hazard ratios (HR) and 95% confidence intervals (CI). Age was adjusted in accordance with epidemiological guidelines recommended by Pearce [18]. Additional covariates included BMI, smoking status (current or former), alcohol intake, and the use of medications reflecting underlying cardiovascular risk, namely antiplatelets, beta-blockers, calcium channel blockers, ACE inhibitors, angiotensin II receptor blockers, and treatments for diabetes and hypercholesterolemia. These variables, detailed in MACEPred dataset section, were selected for their established role in cardiovascular risk prediction and as proxies for conventional risk factors such as hypertension, dyslipidemia, and insulin resistance.

For comparability with previous studies [11, 19], age and BMI were treated as continuous variables in the primary analysis. A sensitivity analysis using categorical versions of age and BMI is provided in the Supplementary Materials. A *p* value of < 0.05 was considered statistically significant.

Model discrimination was additionally evaluated using time-fixed receiver operating characteristic (ROC) analysis at predefined follow-up horizons 5 years. Predicted event risks were derived from the Cox models, and areas under the ROC curve (AUCs) were calculated to compare the discriminative performance of models including traditional risk factors alone versus models incorporating BAC presence and BAC grading.

## Results

### Performance of the segmentation model

**Fig. 2:**
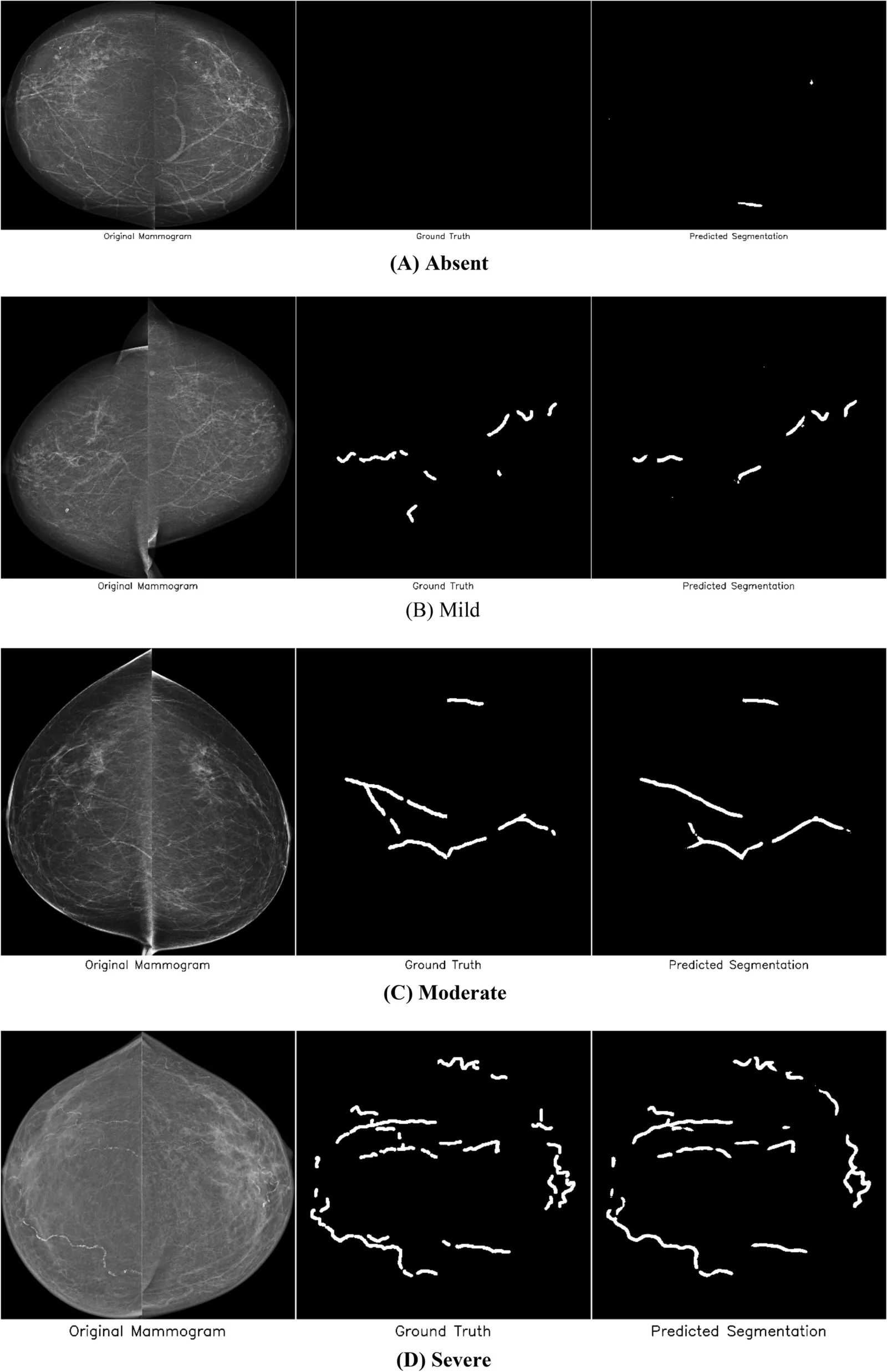
Representative examples of automated breast arterial calcification (BAC) segmentation across severity grades: **A** absent, **B** mild, **C** moderate, and **D** severe. For each case, original mammogram, ground-truth annotation, and model-predicted segmentation are shown

The U-Net model achieved a Jaccard Similarity Coefficient of 0.582, accuracy of 0.996, precision of 0.801, F1 score of 0.756, and recall of 0.716, indicating strong performance in segmenting breast arterial calcifications (BAC), a feature that typically occupies a very small proportion of the mammographic image. For reference, SCU-Net, as reported by Guo et al. [12], achieved a Jaccard index of 0.581, accuracy of 0.997, precision of 0.708, F1 score of 0.729, and recall of 0.789 when evaluated on full-size mammograms. Although direct comparisons are limited by differences in datasets and evaluation protocols, the reported performance of our model is consistent with previously published results. Figure 2A–D illustrates representative segmentation outputs across BAC severity grades, showing the predicted regions alongside the corresponding ground-truth annotations.

### Binary categorization of breast arterial calcification

In the MACEPred dataset, the prevalence of BAC was found to be 26.96%, a figure consistent with existing literature for this age group [8], thereby confirming the representativeness of our study population. Initially, the Cox proportional hazard model, adjusted solely for age, evaluated the risk of MACE associated with the presence of BAC, defined by a threshold indicating BAC coverage of 0.2% or more of the breast area in mammograms. This model yielded an HR of 1.16 (95% CI 1.10–1.23) for the presence of BAC above this threshold, indicating a 16% increased hazard for MACE when BAC is present. Subsequent adjustments for other CVD risk factors did not alter the significance of this finding, maintaining the HR at the same value, as detailed in Table 2.

**Table 2:** Hazard ratios (HRs) for cardiovascular disease (CVD) risk factors when using binary classification of cases based on the presence of breast arterial calcifications (BAC). The *p* values for all risk factors are < 0.05

Characteristic	Case (*N* = 3190)	Control (*N* = 6458)	HR	%95 CI
Age	63.2 ± 7.6 ^x̄^	63.9 ± 7.9 ^x̄^	1.006	(1.00–1.01)***
BMI	26.5 ± 9.9 ^x̄^	25.6 ± 8.3 ^x̄^	1.003	(1.00–1.01)*
If had a drink last year	2463 (77%)	5262 (81%)	0.875	(0.82–0.93)***
Being a current smoker	255 (8%)	287 (4%)	1.845	(1.68–2.03)***
Being an ex-smoker	1172 (37%)	2271 (35%)	1.228	(1.16–1.30)***
Presence of BAC	949 (30%)	1652 (26%)	1.164	(1.10–1.23)***
*Medications usage*				
Medications for diabetes	321 (10%)	280 (4%)	1.267	(1.16–1.38)***
Medications for high cholesterol	1252 (39%)	1784 (28%)	1.128	(1.07–1.20)***
Beta blockers	284 (9%)	240 (4%)	1.463	(1.34–1.60)***
Calcium channel antagonists	360 (11%)	409 (6%)	1.112	(1.03–1.21)**
ACE inhibitors	754 (24%)	921 (14%)	1.187	(1.12–1.26)***
Angiotensin II antagonists	825 (26%)	1070 (17%)	1.178	(1.11–1.25)***
Antiplatelets	981 (31%)	936 (14%)	1.578	(1.49–1.68)***

^*^p value < 0.05 and > 0.01

^**^p value < 0.01 and > 0.001

^***^p value < 0.001

### Breast arterial calcification severity grading

Table 3 shows the adjusted HRs for various grading categories for area-based, intensity-based and combined approaches. As shown, when considering the area-based approach, after incorporating all available CVD risk factors adjusted HRs for mild, moderate, and severe BAC grades were determined to be 1.13 (95% CI 1.06–1.20), 1.30 (95% CI 1.18–1.45), and 1.58 (95% CI 1.15–2.18), respectively. For intensity-based grading, with BAC “absent” serving as the reference category, the model yielded HRs of 1.08 (95% CI 1.00–1.17) for mild, 1.10 (95% CI 1.01–1.20) for moderate, and 1.18 (95% CI 1.09–1.28) for severe. This consistent trend across BAC intensity grades suggests the potential of BAC intensity as an independent predictor of cardiovascular risk.

**Table 3:** Hazard ratios (HRs) for cardiovascular disease (CVD) risk factors when using the area and intensity-based grading system for quantifying the presence and severity of the breast arterial calcifications (BAC). The *p* values for all risk factors are < 0.05. 95% confidence intervals (CI) are also presented

Characteristic	Area-based	Intensity-based	Combined	
	HR 95% CI	HR 95% CI	HR	95% CI
Age	1.005 (1.00–1.01)**	1.006 (1.00–1.01)****	1.006 (1.00–1.01)**	
BMI	1.003 (1.00–1.01)*	1.003 (1.00–1.01)	1.003 (1.00–1.01)*	
If had a drink last year	0.875 (0.82–0.93)***	0.875 (0.82–0.93)***	0.875 (0.82–0.93)***	
Being a current smoker	1.847 (1.68–2.03)***	1.849 (1.68–2.03)***	1.854 (1.68–2.03)***	
Being an ex-smoker	1.232 (1.17–1.30)***	1.227 (1.16–1.29)***	1.233 (1.16–1.29)***	
BAC grading: mild	1.126 (1.06–1.20)***	1.077 (1.00–1.17)¶	1.102 (1.04–1.17)**	
BAC grading: moderate	1.304 (1.18–1.45)***	1.099 (1.01–1.20)*	1.249 (1.14–1.37)***	
BAC grading: severe	1.584 (1.15–2.18)**	1.183 (1.09–1.28)***	1.654 (1.30–2.10)***	
*Medications usage*				
Medications for diabetes	1.264 (1.16–1.40)***	1.263 (1.16–1.38)***	1.265 (1.16–1.38)***	
Medications for high cholesterol	1.129 (1.07–1.19)***	1.127 (1.07–1.19)***	1.128 (1.07–1.19)***	
Beta blockers	1.461 (1.34–1.60)***	1.457 (1.33–1.59)***	1.458 (1.33–1.59)***	
Calcium channel antagonists	1.109 (1.02–1.20)***	1.109 (1.02–1.20)*	1.108 (1.02–1.20)*	
ACE inhibitors	1.187 (1.12–1.26)*	1.185 (1.11–1.26)***	1.185(1.11–1.26)***	
Angiotensin II antagonists	1.178 (1.11–1.25)***	1.175 (1.11–1.25)***	1.176 (1.11–1.25)***	
Antiplatelets	1.577 (1.49–1.68)***	1.578 (1.49–1.68)***	1.576 (1.49–1.68)***	

*p* value < 0.1 and > 0.05

^*^p value < 0.05 and > 0.01

^**^p value < 0.01 and > 0.001

^***^p value < 0.001

When the grading strategies were combined into a unified approach, the adjusted HRs for mild, moderate, and severe BAC grades were 1.102 (95% CI 1.04–1.17), 1.249 (95% CI 1.14–1.37), and 1.654 (95% CI 1.30–2.10), respectively. These results indicate that the combined grading approach may offer enhanced risk stratification compared to area- or intensity-based grading alone.

### Discriminative performance of BAC-augmented models

**Fig. 3:**
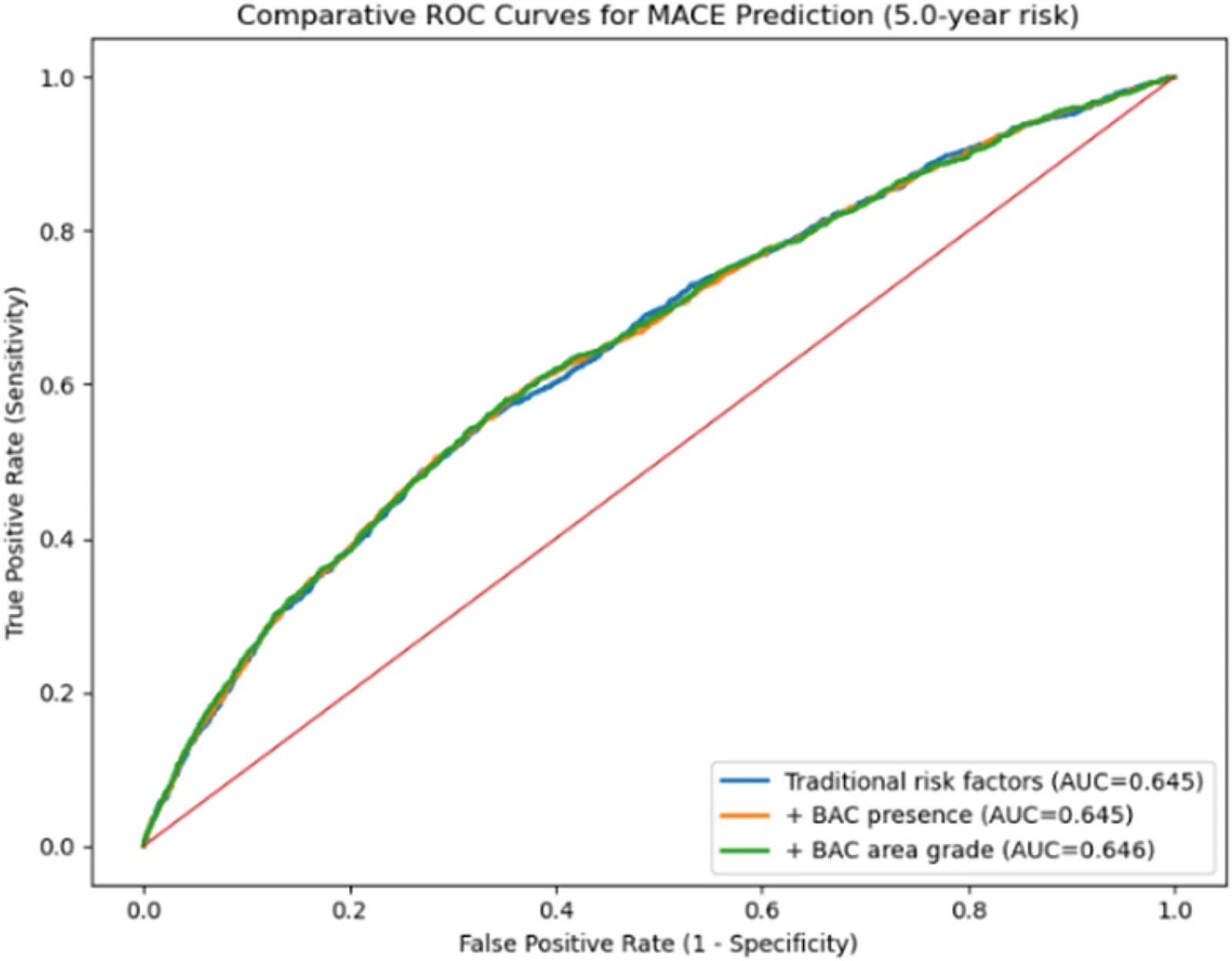
Comparative receiver operating characteristic (ROC) curves for prediction of major adverse cardiovascular events (MACE) at 5-year follow-up using traditional cardiovascular risk factors alone, with the addition of BAC presence, and with BAC area-based grading

At the 5-year follow-up horizon, the model including traditional cardiovascular risk factors demonstrated moderate discrimination for MACE prediction (AUC = 0.645). The addition of BAC presence did not materially change model discrimination (AUC = 0.645), while incorporation of BAC area grade resulted in a minimal numerical increase in AUC (AUC = 0.646) (Fig. 3). Overall, ROC curves for the three models showed substantial overlap, indicating similar discriminative performance at this time point.

## Discussions

Our study proposed a deep learning-based automated solution for segmenting BAC on mammograms from multiple vendors and translating the segmented areas into an easy-to-communicate, independent grading system. Our results show an association between BAC and increased risk for extended MACE, aligning with several previous studies [11, 20–22] that examined the relationship between BAC and cardiovascular events. Training and validating our model on a multi-vendor dataset likely contributed to improved generalizability and robustness. Compared to SCU-Net, a previously published model by Guo et al. [12], our model achieved higher performance in Jaccard similarity and F1 score, which are particularly important for segmenting small and sparse structures such as BAC. When present, BAC typically occupies less than 1–2% of the breast area, making segmentation inherently challenging and limiting the interpretability of overall accuracy in this context. Differences in image preprocessing approaches, such as the use of full-size versus breast-cropped images, may also influence performance metrics. As a result, overlap-based metrics such as the Jaccard index and F1 score provide a more informative evaluation of segmentation quality.

A formal radiologist validation study of the BAC segmentation framework (including interobserver agreement using weighted κ) is reported in our related segmentation-focused publication [17]. The present study builds on that validated segmentation approach to assess the relationship between automated BAC grading (area and intensity) and linked cardiovascular outcomes.

Our study explores multiple grading strategies (binary, area-based, intensity-based, and combined). Unlike Wang et al. [13] and AlGhamdi et al. [14], who focused only on binary BAC detection, our model categorizes BAC into “mild,” “moderate,” and “severe.” Similar to the coronary artery calcium (CAC) score, which employs categories such as absent, minimal, mild, moderate, and extensive [19, 20] and indicates nonlinear increments in HRs for cardiovascular events [21, 22]. Similarly, our data reveal progressive and nonlinear increases in HRs with BAC severity across all grading strategies. Area-based grading exhibited a more pronounced dose–response association than intensity-based grading, with hazard ratios increasing from 1.13 to 1.58 across severity categories, compared with a smaller increase from 1.08 to 1.18 for intensity-based grading (Table 3). Notably, the combined area- and intensity-based approach showed the clearest separation between risk groups, with hazard ratios progressively increasing from 1.10 for mild to 1.65 for severe BAC.

In Iribarren et al.’s study 

[11], BAC was linked to a 1.51-fold heightened risk of atherosclerotic cardiovascular disease and a marginally increased HR of 1.23 for global CVD over a mean 6.5-year follow-up. For those with BAC > 0, severity was categorized into tertiles. However, their study did not conclusively demonstrate a dose–response association with global CVD, possibly as a result of limiting the scope to postmenopausal women and did not encompass specific CVD events such as myocardial infarction. Furthermore, the analysis did not evaluate the number of calcified vessels, which correlates with the BAC coverage area.

The proposed grading system may complement traditional cardiovascular risk models, which often underestimate risk in women, by providing an independent imaging-based marker of vascular calcification. As BAC grading can be derived opportunistically from routine mammography without additional imaging or procedural burden, it offers a practical means for incorporating cardiovascular risk information into breast screening workflows, particularly for asymptomatic women. In a clinical setting, the automated system could operate as a background analysis within existing PACS infrastructure, returning BAC grades as structured reports or visual overlays without perceptible impact on routine image interpretation. Automated grading may support earlier identification of high-risk individuals and facilitate timely referrals. A standardized framework could also support the formal inclusion of BAC assessment in clinical guidelines and improve consistency in cardiovascular risk reporting for women.

It is important to acknowledge several limitations of this study. First, direct measurements of conventional cardiovascular risk factors such as blood pressure, diabetes status, or serum cholesterol levels were not available. Instead, medication use was employed as a surrogate marker for these conditions, which may introduce misclassification. Future studies incorporating direct clinical measurements could provide additional insights, particularly as linkage to routinely collected health records is feasible and would not incur additional costs when implementing the proposed framework. Second, this retrospective study focused on women with a mean age of 63 years; therefore, the generalizability of the findings to younger or older populations may be limited. However, as the cohort was derived from a population-based screening archive, women outside the standard screening age range were inherently underrepresented. Third, the analysis was restricted to a single screening round; consequently, longitudinal changes in BAC burden could not be assessed, and the association between BAC progression (e.g., from mild to severe) and subsequent MACE risk could not be evaluated. Longitudinal studies are warranted to investigate temporal changes in BAC and their relationship with cardiovascular outcomes over time. In addition, although the training and evaluation datasets included images from multiple mammography manufacturers, segmentation performance was not stratified by imaging system. Future work will assess potential vendor-specific differences in model performance. Finally, extending the analysis to include radiomic features and more detailed BAC characteristics beyond area and intensity may further refine cardiovascular risk assessment.

The linked MACE outcome used in this study represents a composite cardiovascular endpoint designed for population-level data linkage and is broader than some clinically conventional “hard MACE” definitions restricted to myocardial infarction, stroke, cardiovascular death, and selected procedures or heart failure. While this approach increases sensitivity to clinically relevant cardiovascular morbidity, it limits evaluation of associations for individual event subtypes because event-level component data were not available in the approved extract. Furthermore, event counts for several individual components would be expected to be lower, limiting power and precision for subtype-specific analyses. Future work will prioritize adjudicated and event-specific outcomes, where event-level subtype data and adequate sample sizes are available.

In conclusion, this study presents a novel framework that automates the detection and quantification of BAC, providing accessible and independent assessment of CVD risk in women. Our model's effectiveness was affirmed through testing across diverse mammography unit types and brands, with the inclusion of a new quantification method enhancing its clinical viability. Importantly, using the proposed grading system, as BAC severity increased, so did the HRs, indicating a significant rise in risk parallel to the increase in BAC severity and intensity. This underscores the potential of BAC as a valuable independent factor for predicting cardiovascular risk in women.

## Supplementary Information

Below is the link to the electronic supplementary material.

Supplementary file1 (DOCX 36 kb)

Supplementary file2 (PNG 227 kb)

Supplementary file3 (DOCX 30 kb)

## Data Availability

Data related to this study will be made available upon reasonable request. Access to the de-identified dataset used for the analysis may be granted with proper institutional and ethical approvals.
