# Supplementary material for: A novel deep learning-based grading system for assessing breast arterial calcification on mammograms, as an independent risk factor for predicting adverse cardiovascular events
Source: Radiol Med. 2026 Jun 29;131(9):1497–507. doi: 10.1007/s11547-026-02251-8 (PMC13658297; doi:10.1007/s11547-026-02251-8)
Supplement: Supplementary file 1 [file 11547_2026_2251_MOESM1_ESM.docx]

# Supplementary Table S1. Sensitivity analysis for BAC presence threshold selection

| Normalized BAC area threshold | Hazard Ratio (HR) | 95% CI (Lower) | 95% CI (Upper) | p-value | N | BAC-positive (n) |
| --- | --- | --- | --- | --- | --- | --- |
| 0.0005 | 1.141 | 1.059 | 1.229 | 0.00052 | 9648 | 5995 |
| 0.00075 | 1.169 | 1.087 | 1.256 | <0.001 | 9648 | 5050 |
| 0.001 | 1.147 | 1.067 | 1.233 | 0.0002 | 9648 | 4349 |
| 0.00125 | 1.156 | 1.073 | 1.244 | 0.00012 | 9648 | 3728 |
| 0.0015 | 1.158 | 1.073 | 1.249 | 0.00015 | 9648 | 3238 |
| 0.00175 | 1.185 | 1.096 | 1.28 | <0.001 | 9648 | 2880 |
| 0.002 | 1.185 | 1.094 | 1.283 | <0.001 | 9648 | 2601 |
| 0.00225 | 1.167 | 1.075 | 1.266 | 0.00023 | 9648 | 2388 |
| 0.0025 | 1.17 | 1.076 | 1.273 | 0.00025 | 9648 | 2210 |
| 0.00275 | 1.16 | 1.064 | 1.264 | 0.00073 | 9648 | 2064 |
| 0.003 | 1.137 | 1.04 | 1.242 | 0.00453 | 9648 | 1934 |
| 0.00325 | 1.136 | 1.038 | 1.243 | 0.00571 | 9648 | 1819 |
| 0.0035 | 1.146 | 1.046 | 1.257 | 0.0036 | 9648 | 1732 |
| 0.00375 | 1.139 | 1.037 | 1.251 | 0.00642 | 9648 | 1633 |
| 0.004 | 1.16 | 1.055 | 1.276 | 0.00219 | 9648 | 1561 |
| 0.005 | 1.221 | 1.102 | 1.351 | 0.00012 | 9648 | 1273 |
| 0.0075 | 1.235 | 1.097 | 1.39 | 0.00049 | 9648 | 849 |
| 0.01 | 1.299 | 1.131 | 1.493 | 0.00021 | 9648 | 575 |
| 0.015 | 1.364 | 1.122 | 1.657 | 0.00179 | 9648 | 249 |
| 0.02 | 1.404 | 1.042 | 1.893 | 0.0259 | 9648 | 94 |
| 0.025 | 1.592 | 1.022 | 2.481 | 0.0398 | 9648 | 38 |
| 0.03 | 1.734 | 0.864 | 3.48 | 0.121 | 9648 | 15 |
