# Supplementary material for: A novel deep learning-based grading system for assessing breast arterial calcification on mammograms, as an independent risk factor for predicting adverse cardiovascular events
Source: Radiol Med. 2026 Jun 29;131(9):1497–507. doi: 10.1007/s11547-026-02251-8 (PMC13658297; doi:10.1007/s11547-026-02251-8)
Supplement: Supplementary file 2 [file 11547_2026_2251_MOESM3_ESM.docx]

**Supplementary Table 1. ICD-10 code definitions of Extended Major Adverse cardiovascular events and components**

| ICD-10 code | Description |
| --- | --- |
| I20* | Angina pectoris |
| I20* | Angina pectoris |
| I21* | Acute myocardial infarction |
| I21* | Acute myocardial infarction |
| I22* | Subsequent myocardial infarction |
| I22* | Subsequent myocardial infarction |
| I23* | Certain current complications following acute myocardial infarction |
| I23* | Certain current complications following acute myocardial infarction |
| I24* | Other acute ischaemic heart diseases |
| I24* | Other acute ischaemic heart diseases |
| I25* | Chronic ischaemic heart disease |
| I25* | Chronic ischaemic heart disease |
| I46* | Cardiac arrest |
| I50* | Heart failure |
| I50* | Heart failure |
| I63* | Cerebral infarction |
| I63* | Cerebral infarction |
| I64* | Stroke, not specified as haemorrhage or infarction |
| I64* | Stroke, not specified as haemorrhage or infarction |
| I65* | Occlusion and stenosis of precerebral arteries, not resulting in cerebral infarction |
| I65* | Occlusion and stenosis of precerebral arteries, not resulting in cerebral infarction |
| I66* | Occlusion and stenosis of cerebral arteries, not resulting in cerebral infarction |
| I66* | Occlusion and stenosis of cerebral arteries, not resulting in cerebral infarction |
| I70* | Atherosclerosis |
| I70* | Atherosclerosis |
| I71* | Aortic aneurysm and dissection |
| I71* | Aortic aneurysm and dissection |
| I72* | Other aneurysm and dissection |
| I72* | Other aneurysm and dissection |
| I73* | Other peripheral vascular diseases |
| I73* | Other peripheral vascular diseases |
| I74* | Arterial embolism and thrombosis |
| I74* | Arterial embolism and thrombosis |
| I77* | Other disorders of arteries and arterioles |
| I77* | Other disorders of arteries and arterioles |

ICD-10 Codes defined by International Classification of Diseases, tenth revision (ICD-10) code World Health Organisation [1]

**Table 2: Hazard Ratios (HRs) for cardiovascular disease (CVD) Risk Factors, including categorized BMI and Age, using binary classification of cases based on the presence of Breast Arterial Calcifications (BAC). The p-values for all risk factors are <0.05.**

| **Characteristic** | **Case**  **(N=** **3190)** | **Control**  **(N=** **6458)** | **HR** | **%95 CI** |
| --- | --- | --- | --- | --- |
| **Age Group 1 VS Reference Age** | 1340 (42%) | 2388 (37%) | 1.08 | (0.999 - 1.167) ¶ |
| **Age Group 2 VS Reference Age** | 1186 (37%) | 2661(41%) | 1.17 | (1.078 - 1.27) *** |
| **Age Group 3 VS Reference Age** | 221(07%) | 543(8%) | 1.134 | (1.003 - 1.283) * |
| **Reference Age** | 443 (14%) | 866 (13%) | N/A | N/A |
| **Underweight VS Normal weight** | 292 (9%) | 454 (07%) | 1.259 | (1.146 - 1.383) |
| **Overweight VS Normal weight** | 951(30%) | 2083 (32%) | 1.057 | (0.991 - 1.128) |
| **Obese VS Normal weight** | 1048 (33%) | 1511 (23%) | 1.212 | (1.134 - 1.295) |
| **Reference BMI Normal weight** | 899 (28%) | 2410 (37%) | N/A | N/A |
| **If had a drink last year** | 2463 (77%) | 5262 (81%) | 0.875 | (0.82 - 0.93) *** |
| **Being a current smoker** | 255 (8%) | 287 (4%) | 1.845 | (1.68- 2.03) *** |
| **Being an ex-smoker** | 1172 (37%) | 2271 (35%) | 1.228 | (1.16 - 1.30) *** |
| **Presence of BAC** | 949 (30%) | 1652 (26%) | 1.164 | (1.10 - 1.23) *** |
| **Medications usage** |  |  |  |  |
| **Medications for Diabetes** | 321 (10%) | 280 (4%) | 1.22 | (1.116 - 1.333) *** |
| **Medications for High cholesterol** | 1252 (39%) | 1784 (28%) | 1.131 | (1.069 - 1.196) *** |
| **Beta blockers** | 284 (9%) | 240 (4%) | 1.461 | (1.336 - 1.597) *** |
| **Calcium channel antagonists** | 360 (11%) | 409 (6%) | 1.111 | (1.025 - 1.204) * |
| **ACE inhibitors** | 754 (24%) | 921 (14%) | 1.17 | (1.1 - 1.244) *** |
| **Angiotensin II antagonists** | 825 (26%) | 1070 (17%) | 1.155 | (1.089 - 1.225) *** |
| **Antiplatelets** | 981 (31%) | 936 (14%) | 1.562 | (1.471 - 1.658) *** |

¶ p-value<0.1 and >0.05

* p-value<0.05 and >0.01

** p-value<0.01 and >0.001

*** p-value<0.001

**Age Categories:**

- **Reference Age**: Age less than 55 years.
- **Age Group 1**: Age between 55 and 64 years.
- **Age Group 2**: Age between 65 and 74 years.
- **Age Group 3**: Age 75 years and above

**BMI Categories:**

- **Underweigh**t: BMI less than 18.5.
- **Normal weight**: BMI between 18.5 and 24.9.
- **Overweight**: BMI between 25 and 29.9.
- **Obese: BMI** 30 and above

**Table 3: Hazard Ratios (HRs) for cardiovascular disease (CVD) Risk Factors when using the area-based and intensity grading system for quantifying the presence and severity of Breast Arterial Calcifications (BAC). The p-values for all risk factors are <0.05. 95% Confidence Intervals (CI) are also presented. This includes both intensity and area in the same model.**

| **Characteristic** | **HR** | **95% CI** |
| --- | --- | --- |
| **Age** | 1.006 | (1.002 - 1.009) ** |
| **BMI** | 1.003 | (1.001 - 1.006) * |
| **If had a drink last year** | 0.874 | (0.823 - 0.928) *** |
| **Being a current smoker** | 1.842 | (1.676 - 2.025) *** |
| **Being an ex-smoker** | 1.231 | (1.167 - 1.299) *** |
| **BAC Grading Area: Mild** | 1.177 | (1.065 - 1.3) ** |
| **BAC Grading Area: Moderate** | 1.435 | (1.211 - 1.701) *** |
| **BAC Grading Area: Severe** | 1.747 | (1.234 - 2.472) ** |
| **BAC Grading Intensity: Mild** | 1.003 | (0.916 - 1.099) ¶ |
| **BAC Grading Intensity: Moderate** | 0.947 | (0.838 - 1.071) ¶ |
| **BAC Grading Intensity: Severe** | 0.903 | (0.78 - 1.046) ¶ |
| **Medications usage** |  |  |
| **Medications for Diabetes** | 1.266 | (1.159 - 1.382) *** |
| **Medications for High cholesterol** | 1.129 | (1.067 - 1.194) *** |
| **Beta blockers** | 1.463 | (1.338 - 1.599) *** |
| **Calcium channel antagonists** | 1.11 | (1.024 - 1.203) * |
| **ACE inhibitors** | 1.188 | (1.118 - 1.263) *** |
| **Angiotensin II antagonists** | 1.179 | (1.112 - 1.25) *** |
| **Antiplatelets** | 1.58 | (1.488 - 1.677) *** |

¶ p-value<0.1 and >0.05

* p-value<0.05 and >0.01

** p-value<0.01 and >0.001

*** p-value<0.001

**Table 4: Hazard Ratios (HRs) for cardiovascular disease (CVD) Risk Factors when using the maximum value of area and intensity for quantifying the presence and severity of Breast Arterial Calcifications (BAC). The p-values for all risk factors are <0.05. 95% Confidence Intervals (CI) are also presented.**

| **Characteristic** | **HR** | **95% CI** |
| --- | --- | --- |
| Age | 1.006 | (1.002 - 1.009) *** |
| BMI | 1.003 | (1.001 - 1.006) * |
| If had a drink last year | 0.875 | (0.824 - 0.93) *** |
| Being a current smoker | 1.848 | (1.681 - 2.031) *** |
| Being an ex-smoker | 1.227 | (1.163 - 1.294) *** |
| BAC Grading Combined: Mild | 1.097 | (1.02 - 1.181) * |
| BAC Grading Combined: Moderate | 1.108 | (1.018 - 1.205) * |
| BAC Grading Combined: Severe | 1.191 | (1.097 - 1.292) *** |
| Medications usage |  |  |
| Medications for Diabetes | 1.262 | (1.156 - 1.378) *** |
| Medications for High cholesterol | 1.127 | (1.066 - 1.192) *** |
| Beta blockers | 1.458 | (1.333 - 1.594) *** |
| Calcium channel antagonists | 1.109 | (1.022 - 1.202) * |
| ACE inhibitors | 1.185 | (1.115 - 1.26) *** |
| Angiotensin II antagonists | 1.175 | (1.109 - 1.246) *** |
| Antiplatelets | 1.58 | (1.486 - 1.676) *** |

* p-value<0.05 and >0.01

** p-value<0.01 and >0.001

*** p-value<0.001
